# Comprehensive analysis of autophagy‐related prognostic genes in breast cancer

**DOI:** 10.1111/jcmm.15551

**Published:** 2020-07-02

**Authors:** Jianguo Lai, Bo Chen, Hsiaopei Mok, Guochun Zhang, Chongyang Ren, Ning Liao

**Affiliations:** ^1^ Department of Breast Cancer, Cancer Center Guangdong Provincial People’s Hospital and Guangdong Academy of Medical Sciences Guangzhou China

**Keywords:** autophagy, breast cancer, gene, model, prognosis

## Abstract

Accumulating evidence revealed that autophagy played vital roles in breast cancer (BC) progression. Thus, the aim of this study was to investigate the prognostic value of autophagy‐related genes (ARGs) and develop a ARG‐based model to evaluate 5‐year overall survival (OS) in BC patients. We acquired ARG expression profiling in a large BC cohort (N = 1007) from The Cancer Genome Atlas (TCGA) database. The correlation between ARGs and OS was confirmed by the LASSO and Cox regression analyses. A predictive model was established based on independent prognostic variables. Thus, time‐dependent receiver operating curve (ROC), calibration plot, decision curve and subgroup analysis were conducted to determine the predictive performance of ARG‐based model. Four ARGs (ATG4A, IFNG, NRG1 and SERPINA1) were identified using the LASSO and multivariate Cox regression analyses. A ARG‐based model was constructed based on the four ARGs and two clinicopathological risk factors (age and TNM stage), dividing patients into high‐risk and low‐risk groups. The 5‐year OS of patients in the low‐risk group was higher than that in the high‐risk group (*P* < 0.0001). Time‐dependent ROC at 5 years indicated that the four ARG–based tool had better prognostic accuracy than TNM stage in the training cohort (AUC: 0.731 vs 0.640, *P* < 0.01) and validation cohort (AUC: 0.804 vs 0.671, *P* < 0.01). The mutation frequencies of the four ARGs (ATG4A, IFNG, NRG1 and SERPINA1) were 0.9%, 2.8%, 8% and 1.3%, respectively. We built and verified a novel four ARG–based nomogram, a credible approach to predict 5‐year OS in BC, which can assist oncologists in determining effective therapeutic strategies.

## 1 INTRODUCTION

Breast cancer (BC) is the major health burden because of the high rates of morbidity and cancer death among women.^1^, ^2^, ^3^ Although great medical advances in BC screening, diagnosis, and comprehensive therapy, its survival outcome is not entirely satisfactory. Thus, individual treatment strategies of breast cancer still present huge challenges. Currently, therapeutic schemes and prognostic assessment for BC patients are mainly based on the tumour‐node‐metastasis (TNM) staging system and molecular subtypes.^4^, ^5^, ^6^ The TNM staging system has been widely applied for cancer management, but it is unable to achieve individualized survival prediction.^7^, ^8^, ^9^ In addition, some cases at the same TNM stage have distinct survival outcome. Then, many BC patients did not receive optimal treatment strategies and had to face with unfavourable survival prognosis.^10^ Therefore, the identification of novel signatures to improve prognostic assessment and clinically guide treatment decisions is indispensable in routine practice.

During the past decade, genome expression profiling can effectively provide detailed information for survival prediction in cancer patients.^11^, ^12^, ^13^ Moreover, gene biomarkers have been considered as an important tool to predict the prognostic outcome and treatment responses for cancer patients.^14^, ^15^, ^16^ Autophagy, also recognizing as type II programmed cell death, is a vital biological process that keeps the stability of the intracellular environment.^17^, ^18^ Besides, autophagy can degrade and recycle components of aged or damaged organelles to promote rapid growth of cancer cells.^19^ Thus, autophagy‐related proteins are able to regulate tumour growth and progression.^20^ Several studies have investigated the prognostic roles of autophagy‐related gene (ARG) signatures in various tumours.^21^, ^22^, ^23^, ^24^, ^25^ However, little attention has been paid to identify the association between ARGs and overall survival (OS) in BC via high‐throughput expression profiles. The high‐throughput platform made contributions to genomic analysis in medical oncology with huge clinical significances. Thus, identifying a novel ARG signature using high‐throughput expression profile to predict 5‐year OS for BC patients is urgently needed.

Hence, in this study, to improve prognostic evaluation in BC patients, we sought to identify ARG signature from The Cancer Genome Atlas (TCGA) database. A nomogram integrating the prognostic ARGs and clinicopathological risk factors was established to predict 5‐year OS and achieve effective risk stratification for BC patients.

## 2 MATERIALS AND METHODS

### 2.1 Patients and study design

The gene expression profiles and clinical information for BC patients were obtained from the TCGA database. Inclusion criteria were included: (a) patients with invasive BC; (b) complete gene expression profiles and follow‐up information; and (c) survival time more than 1 month. Eventually, 1007 BC patients (training cohort) satisfying the inclusion criteria were selected in this study. Based on the computer allocation numbers, 504 cases as validation set were randomly confirmed from the training set.

We acquired TCGA data from public database (https://cancergenome.nih.gov/), and hence, ethical approval and patient's informed consent were waived in this study.

### 2.2 Development of risk score and the ARG‐based model

The 232 ARGs were acquired from the HADb (Human Autophagy Database, http://autophagy.lu/clustering/index.html). Firstly, univariate Cox proportional hazards regression analysis (CPHRA) was conducted to investigate the association between the expression level of ARGs and 5‐year OS. Next, the ARGs were recognized as significant variables when the *P* value was <0.05 in the univariate CPHRA. Subsequently, the least absolute shrinkage and selection operator (LASSO) regression analysis was performed to screen out ARGs as candidate genes. LASSO regression analysis is able to shrink regression coefficients towards zero. And on the basis of the regulation weight λ, ARGs with the regression coefficients (equal to zero) were excluded. Finally, the multivariate CPHRA was implemented to identify the independent prognostic ARGs for 5‐year OS in BC patients (*P* < 0.05). In the light of the expression values of selected ARGs, the risk score was constructed and weighted by multivariate Cox regression coefficients. The risk score = sum of regression coefficients × expression level of ARGs. Finally, a ARG‐based model, integrating the prognostic ARGs and clinicopathological risk factors, was formulated. According to the ARG‐based model, the prognostic nomogram score was calculated for each patient.

### 2.3 Evaluation of the ARG‐based model

The model accuracy of the ARG‐based tool was estimated using time‐dependent receiver operating characteristic (ROC) curve and the area under the curve (AUC) value. The calibration ability of the ARG‐based model was assessed via the calibration plot. Calibration curve was executed to validate the agreement between nomogram‐predicted and actual outcome. The risk stratification ability of the ARG‐based model was validated by subgroup analysis. Decision curve analysis (DCA) has been advocated to assess the potential clinical utility of predictive nomogram. Herein, DCA was applied to evaluate the clinical usefulness of the model by quantifying the net benefits for a range of threshold probabilities. DCA was executed as an analytical method that combined the results of a decision to quantify the clinical practicability of a model. Thus, the DCA can weigh the net benefit to assess the clinical utility of a nomogram.^26^, ^27^, ^28^, ^29^, ^30^, ^31^

### 2.4 Statistical analysis

Distribution differences in variables in the two independent cohorts were evaluated using the chi‐square and the Mann–Whitney U test. Univariate, LASSO and multivariate Cox regression analyses were performed to identify the independent indicators of OS (*P* < 0.05). Then, Cox regression coefficients were used to build a risk score and four ARG–based nomogram. Time‐dependent ROC curve analysis is extensively carried out to assess the predictive performance of the four ARG–based model. Survival curves were drawn using the Kaplan‐Meier method and contrasted via the log‐rank test. OS (primary end‐point) was calculated as the interval from surgery to the time of death or last follow‐up. We confirmed the optimal cut‐off value of the ARG‐based nomogram using X‐tile plot.^32^, ^33^, ^34^ Statistical analyses were applied via Stata/MP, version 14.0 (StataCorp LP, College Station, TX, USA) and R version (3.4.4, www.r-project.org). *P* < 0.05 was considered as statistically significant.

## 3 RESULTS

### 3.1 Identification of the four ARG signatures

**FIGURE 1:**
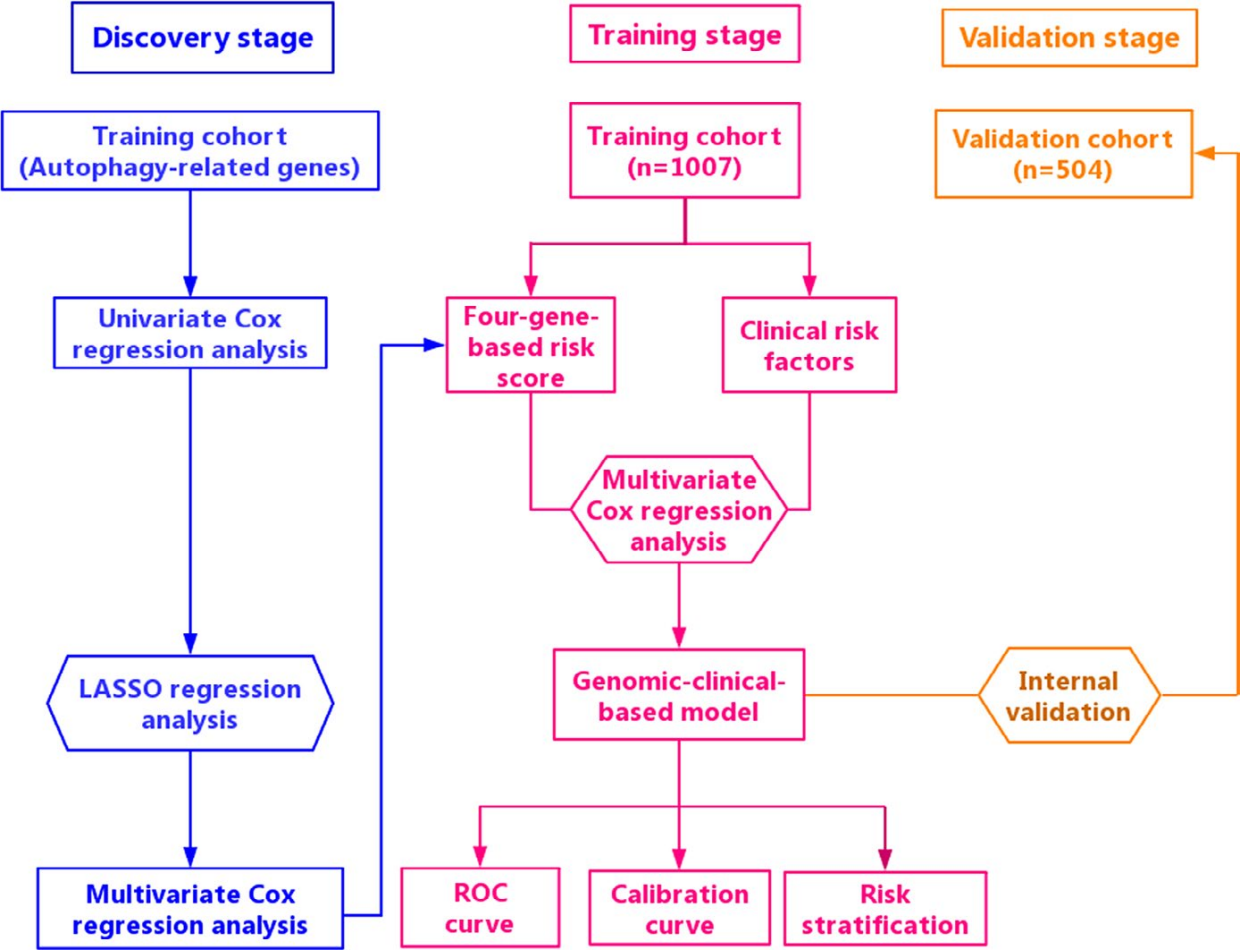
The detailed flow chart of the study procedure

The detailed flow chart of the study procedure is presented in Figure 1. And detailed baseline characteristics of included patients are shown in Table 1. There were no distinct differences in patient characteristics between two independent cohorts in Table 1 (*P* > 0.05). In the light of univariate CPHRA, 39 ARGs were preliminary screen out in the training cohort (*P* < 0.05). Subsequently, LASSO Cox regression analysis was carried out to determine 17 ARGs as candidate genes in the primary data set (Figure S1). To assess the potential function of the 17 prognostic ARGs, functional enrichment analysis was performed via Metascape database (http://metascape.org/). And the result suggested that the 17 ARGs mainly enriched in autophagy, positive regulation of protein localization to membrane, pid delta np63 pathway, glial cell differentiation, regulation of cellular response to heat and establishment of protein localization to organelle (Figure S2). Next, multivariate CPHRA was applied to further identify four ARGs (ATG4A, IFNG, NRG1 and SERPINA1) as independent prognostic indicators in the training cohort. Moreover, we also found that the mutation frequencies of the four ARGs (ATG4A, IFNG, NRG1 and SERPINA1) were 0.9%, 2.8%, 8% and 1.3% in the TCGA database, respectively. The common mutation type of the four ARGs was copy‐number amplification (Figure S3). Some studies have revealed that abnormal DNA methylation is a vital mechanism for epigenetic silencing of gene expression in variety of tumours. Thus, we explored this relationship between the four ARGs and DNA methylation in the TCGA database via the MEXPRESS tool (http://mexpress.be/).^35^ For example, as shown in Figure S4, there was a distinct inverse association between DNA methylation and ATG4A expression in the promoter region located close to the TSS and gene body (*P* < 0.05). However, IFNG expression was positively correlated with DNA methylation in its 3′UTR with (Pearson *r* = +0.336, *P* < 0.001). These results suggested that the multiple DNA methylation regions may play important roles in regulating the four ARG expression.

**TABLE 1:** Baseline characteristics of the included patients

Variables	Training cohort	Validation cohort	*P* value
	No. (%)	No. (%)	
No. of patients	1007	504	
Age (years)	58 (48, 67)	57 (48, 66)	0.360
T stage			
T1	272 (27.0)	135 (26.8)	0.959
T2	573 (56.9)	283 (56.1)	
T3	125 (12.4)	69 (13.7)	
T4	34 (3.4)	16 (3.2)	
Tx	3 (0.3)	1 (0.2)	
N stage			
N0	465 (46.2)	231 (45.8)	0.872
N1	345 (34.3)	166 (32.9)	
N2	109 (10.8)	64 (12.7)	
N3	71 (7.0)	35 (7.0)	
Nx	17 (1.7)	8 (1.6)	
TNM stage			
I	176 (17.5)	231 (45.8)	0.912
II	567 (56.3)	166 (32.9)	
III	224 (22.2)	64 (12.7)	
IV	18 (1.8)	35 (7.0)	
Unknown	22 (2.2)	8 (1.6)	

Abbreviation: TNM, tumour‐node‐metastasis.

### 3.2 Establishment of four ARG–based risk score and prognostic model

**FIGURE 2:**
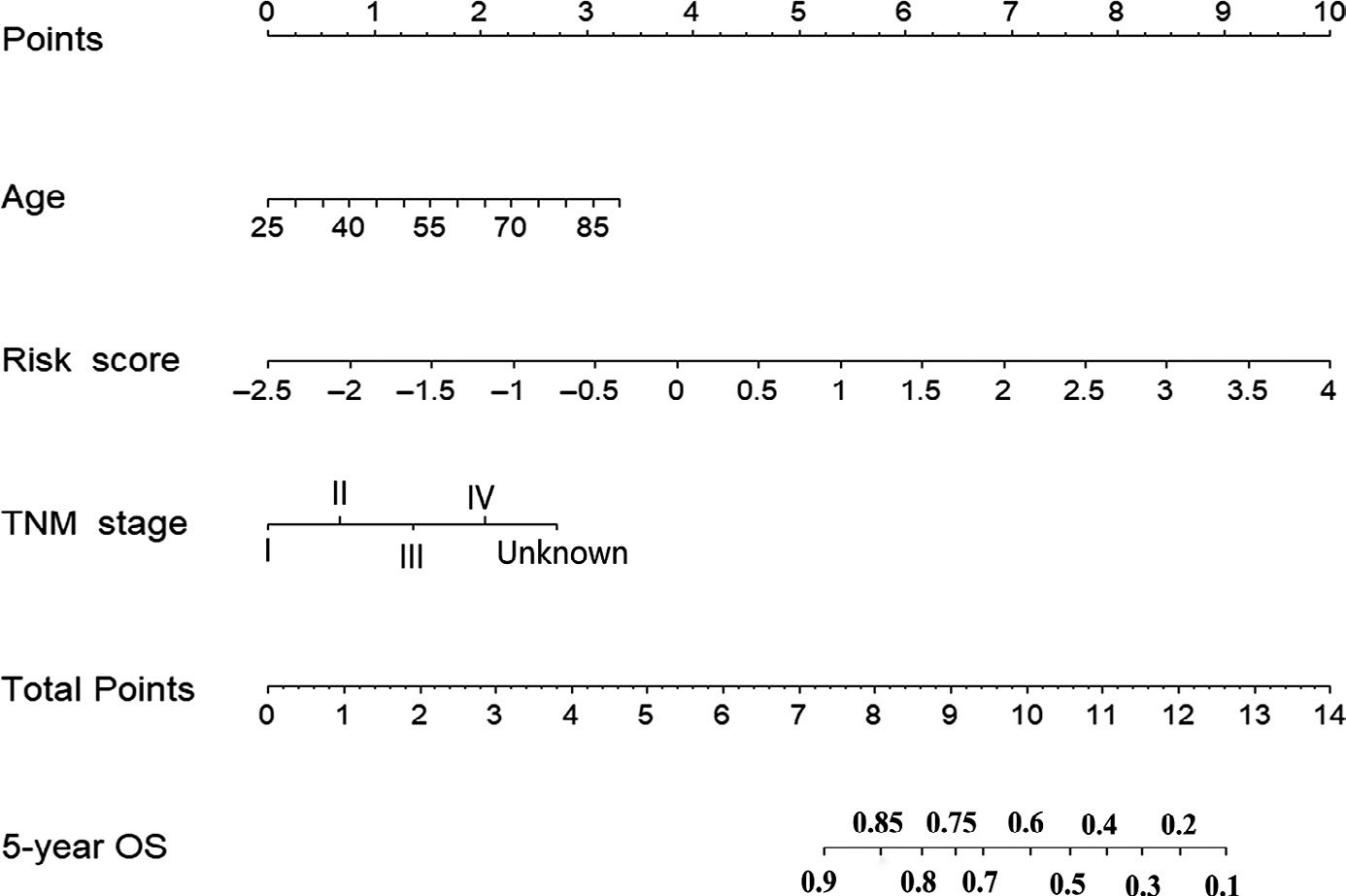
Four ARG–based prognostic model to predict 5‐year OS in breast cancer patients

Using the multivariate Cox regression coefficients, we computed a risk score for each patient; risk score = (0.903 × expression level of ATG4A) + (−0.566 × expression level of IFNG) + (−0.667 × expression level of NRG1) + (−0. 214 × expression level of SERPINA1). The univariate and multivariate CPHRAs in the training set are presented in Table 2. Finally, the risk score and two clinicopathological risk factors (age and TNM stage) were verified as independent predictors for 5‐year OS (*P* < 0.05). Thus, to provide the oncologists with a quantitative technique for predicting the 5‐year OS, we developed a ARG‐based nomogram, which incorporated the four ARG–based risk score and two clinicopathological risk factors (age and TNM stage) (Figure 2).

**TABLE 2:** Univariate and multivariate analyses in the training cohort

Variables	Univariate analysis		Multivariate analysis	
	HR (95% CI)	*P* value	HR (95% CI)	*P* value
Age	1.033 (1.019‐1.047)	**<0.001**	1.028 (1.014‐1.043)	**<0.001**
T stage				
T1	Referent			
T2	1.188 (0.785‐1.799)	0.415		
T3	1.299 (0.744‐2.268)	0.357		
T4	3.414 (1.779‐6.551)	**<0.001**		
Tx	0.571 (0.077‐4.240)	0.584		
N stage				
N0	Referent			
N1	1.819 (1.212‐2.731)	**0.004**		
N2	2.795 (1.633‐4.783)	**<0.001**		
N3	4.136 (2.199‐7.780)	**<0.001**		
Nx	6.506 (3.150‐13.437)	**<0.001**		
TNM stage				
I	Referent		Referent	
II	1.456 (0.838‐ 2.528)	0.183	1.463 (0.840‐2.550)	0.179
III	2.651 (1.473‐4.772)	**0.001**	2.6737 (1.477‐4.841)	**0.001**
IV	11.143 (5.422‐22.901)	**<0.001**	7.3441 (3.555‐15.168)	**<0.001**
Unknown	2.975 (1.333‐ 6.643)	**0.008**	2.9272 (1.306‐ 6.561)	**0.009**
Risk score	2.718 (2.050‐3.604)	**<0.001**	2.373 (1.774‐3.175)	**<0.001**

Note

Bold values indicate statistical significance (*P* < 0.05).

Abbreviations: CI, confidence interval, HR, hazard ratios.

### 3.3 Assessment of the four ARG–based prognostic model

**FIGURE 3:**
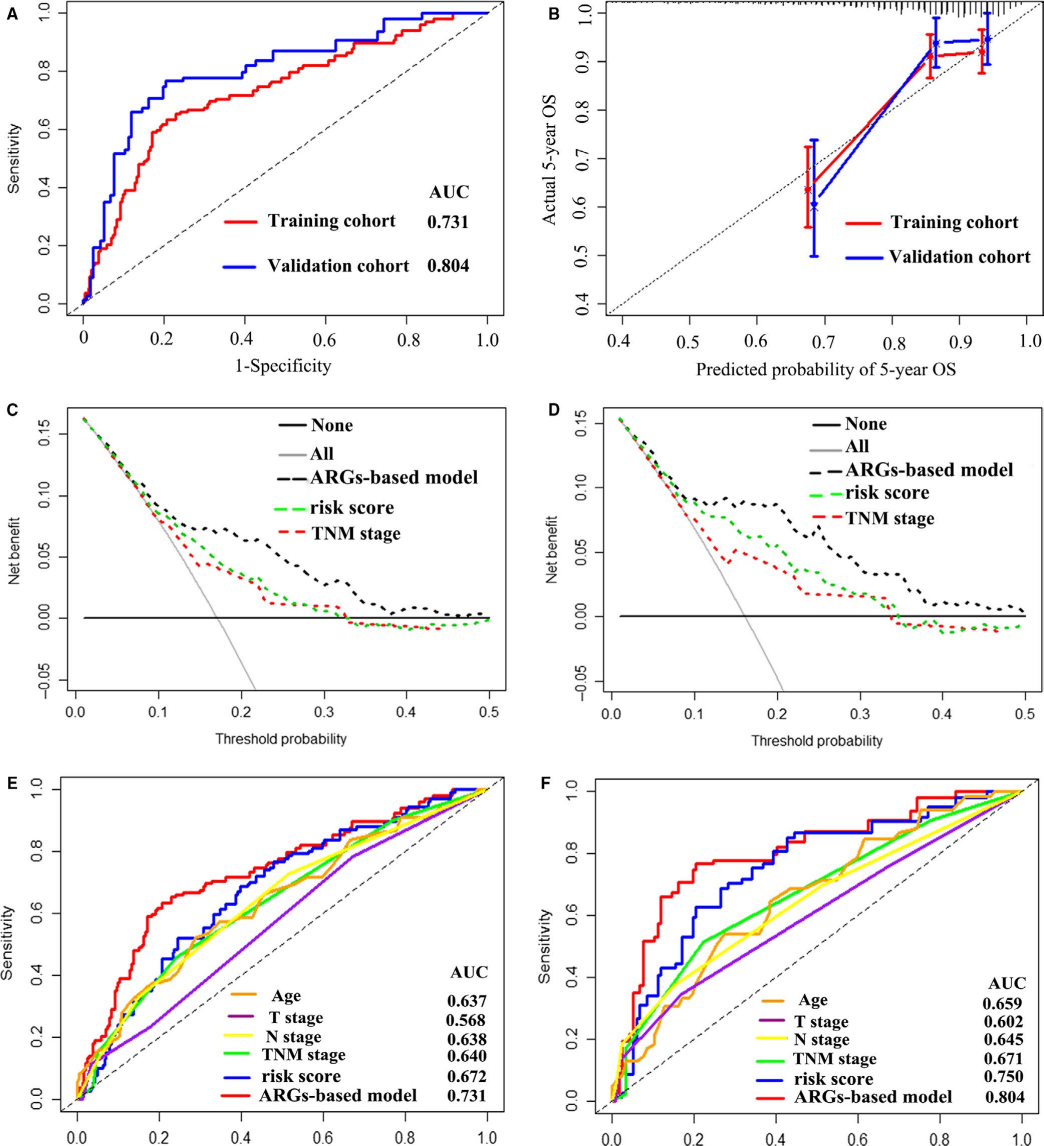
A, Time‐dependent receiver operating characteristic (ROC) curves at 5 y based on the four ARG–based prognostic model in the training cohort and validation cohort. B, Calibration curves of the four ARG–based prognostic model in the training cohort and validation cohort. Decision curve of the four ARG–based prognostic model in the training cohort (C) and validation cohort (D). Comparisons of the predictive accuracy at 5‐y OS using time‐dependent ROC curves in the training cohort (E) and validation cohort (F)

**FIGURE 4:**
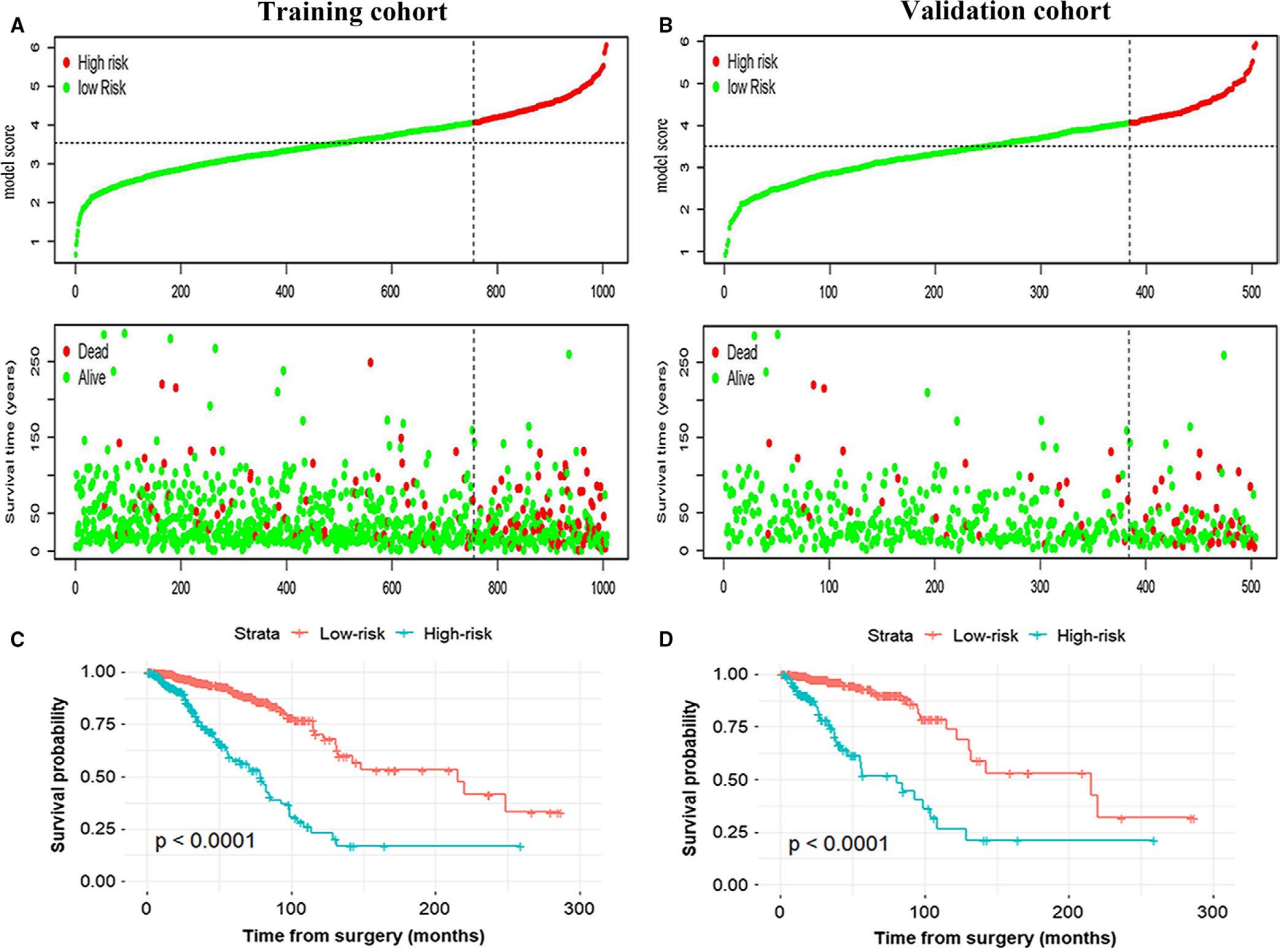
The distribution of model score, OS and OS status in the training cohort (A) and validation cohort (B). The dotted line reveals the optimal cut‐off point of the model score to classify patients into the low‐ and high‐risk group. Kaplan‐Meier curves of the low‐ and high‐risk patients based on the four ARG–based prognostic model in the training cohort (C) and validation cohort (D)

**FIGURE 5:**
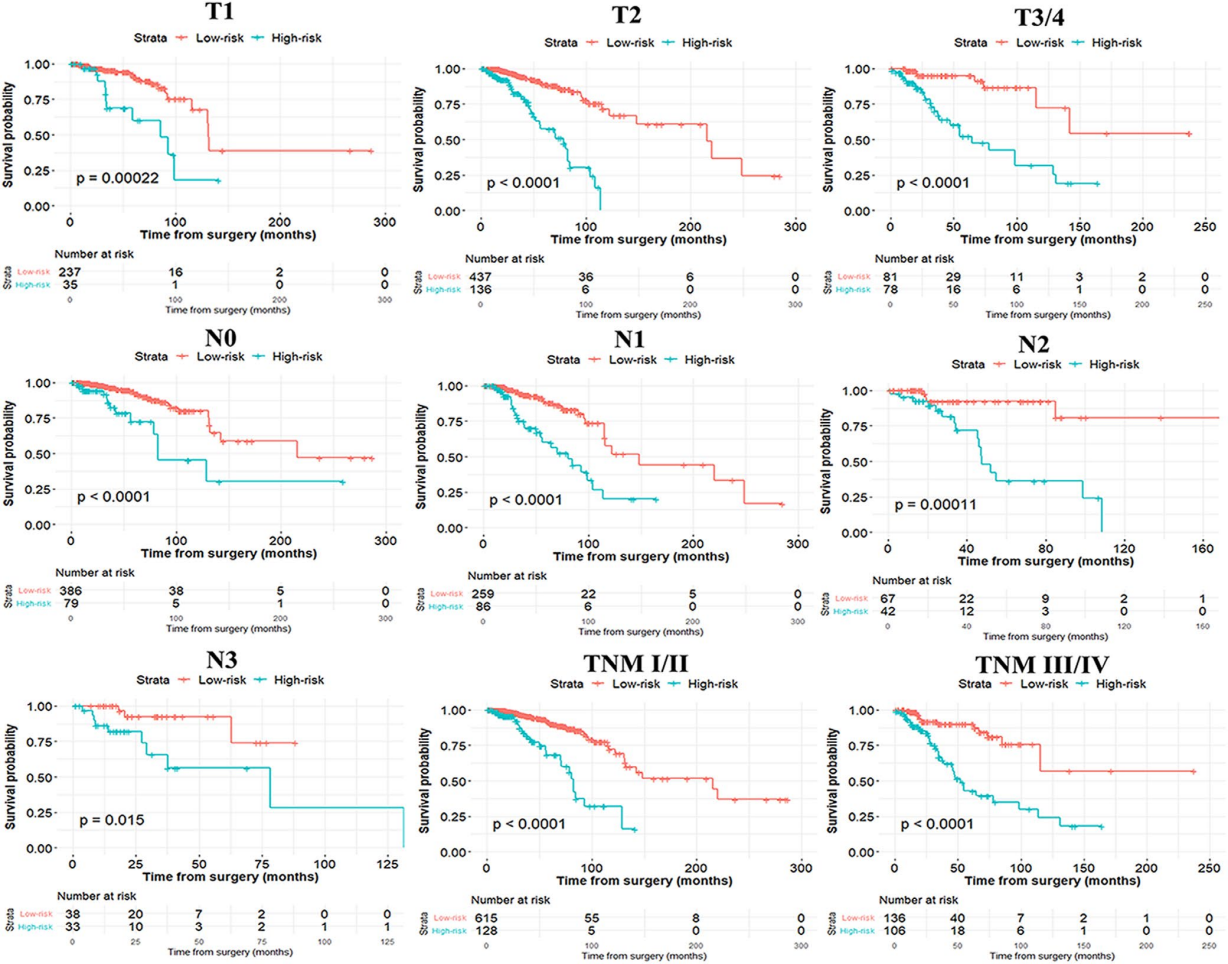
Subgroup analysis of the four ARG–based prognostic model for breast cancer patients in T stage, N stage and TNM stage

The AUC of the four ARG–based model was 0.731 (95% CI: 0.662‐0.801) and 0.804 (95% CI: 0.715‐0.893) in the training and validation groups, indicating that this nomogram had a high predictive accuracy (Figure 3A). Calibration curves indicated great agreement between four ARG–based model‐predicted probabilities and the actual observations of 5‐year OS, showing that this model had good calibration ability (Figure 3B). The DCA demonstrated that the four ARG–based model added more net benefit than the risk score and TNM stage in the training and validation cohorts, suggesting that our model had higher clinical utility (Figure 3C‐D). Besides, to investigate whether the four ARG signatures added additional prognostic value of 5‐year OS, time‐dependent ROC curve was conducted to compare the predictive performances between the ARG‐based nomogram, clinical risk factors and risk score in the training and validation data sets, revealing that prognostic accuracy of the four ARG–based model was superior to risk score and clinical risk factors (Figure 3E‐F). Each patient can be allocated a prognostic score on the basis of the four ARG–based nomogram. Using X‐tile plot, the optimal cut‐off value of the four ARG–based prognostic score was 1.53. Hence, patients were classified into the high‐risk group (N = 252) and low‐risk group (N = 755) in the training cohort. To identify the robustness of the four ARG–based model, it was further tested in the validation data set via the same cut‐off point. Then, BC patients were classified into the low‐risk group (N = 384) and the high‐risk group (N = 120) in the validation cohort. The distribution characteristics of prognostic scores and survival status are manifested in Figure 4, which demonstrated that patients with higher scores had worse OS than that of those with lower scores (*P* < 0.001). Stratified analyses were applied for BC patients in T stage, N stage and TNM stage, suggesting that the four ARG–based model had distinct risk stratification ability (Figure 5). The patients with high‐risk scores had distinctly worse OS than patients with low‐risk scores in T1 (*P* = 0.00022), T2 (*P* < 0.0001), T3/4 (*P* < 0.0001), N0 (*P* < 0.0001), N1 (*P* < 0.0001), N2 (*P* = 0.00011) and N3 (*P* = 0.015), TNM stage I/II (*P* < 0.0001) and TNM stage III/ IV (*P* < 0.0001) (Figure 5).

## 4 DISCUSSION

In the genomic era, novel crucial strategies are imperative to predict survival prognosis and further guide individual treatment for BC patients. Nevertheless, BC progression and survival outcome can vary noticeably owing to the discrepant genes, even though some BC patients are in the identical TNM stage, suggesting the conventional staging technique could not be sufficient for accurate prediction in BC prognosis. In the current circumstances, it is urgent to establish a novel model to accurately predict patients’ survival prognosis. Never before has a study proposed an accurate model taken into account ARGs and clinical features to evaluate the 5‐year OS in BC patients. Accordingly, in consideration of the inherent deficiency of TNM stage system, a novel prognostic model on the basis of the four ARGs (ATG4A, IFNG, NRG1 and SERPINA1) and two clinicopathological factors (age and TNM stage) was constructed to improve individual prediction of 5‐year OS and achieve effective subgroup stratification for BC patients. In terms of predictive accuracy, the AUC of the four ARG–based model distinctly outperformed the current TNM stage system. Moreover, the calibration curves revealed outstanding agreement between the actual and model‐predicted survival probabilities, ensuring the calibration ability of our model. Furthermore, the DCA demonstrated that the four ARG–based nomogram had favourable clinical application across the wider range of threshold probability. In addition, the four ARG–based classifier noticeably stratified BC patients into two risk groups. To our knowledge, this is the first study to perform comprehensive analysis of autophagy‐related prognostic genes in BC. Hence, the novel ARG–based signature could help identify the high‐risk patients and guide individualized treatment of BC patients, which is credible to be applied in clinical application.

In the past decade, accumulating advanced technologies have been exploited to perform transcriptome analysis, including high‐throughput sequence and microarray methods. Recently, many multi–gene‐based biomarkers had been established to predict survival outcome in BC patients.^36^, ^37^, ^38^, ^39^, ^40^, ^41^, ^42^ However, on one hand, some previous studies did not apply LASSO Cox regression model to identify the gene signatures.^36^, ^38^, ^39^ As we known, LASSO has been considerably applied to select optimal variables with a high prognostic ability and avoid overfitting in high‐dimensional data. Therefore, the previous multi–gene‐based models may be not reliable and robust in those studies. On the other hand, growing evidence indicated that autophagy played vital roles in multiple tumour progression, which facilitated us to exploit its importance as prognostic biomarker. Although several researches had investigated the pivotal roles of ARGs in patients' prognosis, the ARG biomarker in BC is still unknown.^21^, ^23^, ^24^ The ATG4A (autophagy‐related 4A cysteine peptidase) is involved in regulating autophagy. Wolf et al reported that ATG4A was imperative for cancer stem cell maintenance and to regulate BC cell tumorigenicity.^43^ And our study elucidated that high expression of ATG4A in BC was associated with poor OS. In concordance with our finding, Yeong et al manifested that high expression of IFNG (interferon gamma) was correlated with favourable survival outcome.^44^ Chan et al demonstrated that high expression of SERPINA1 (serpin family A member 1) may be a predictive indicator for better BC prognosis, which was in accordance with our study.^45^ The role of NRG1 (neuregulin 1) in BC prognosis should be verified. Inevitably, several limitations should be presented in this study. First of all, the established ARG‐based model was stem from the TCGA database. Thus, this nomogram needed to be tested in larger clinical trials with adequate information (adjuvant therapy strategies) in the future. Besides, several crucial clinical risk factors, such as hormone receptor, molecular subtype, adjuvant chemotherapy, ki67 and radiotherapy, were unavailable in the TCGA database. Herein, we are unable to conduct stratification analyses in these subgroup patients. And more biomarkers should be integrated into our model to improve predictive accuracy in the future. Last but not least, some functional experiments on the role of the four ARGs in BC are still needed to be exploited in the future studies.

In conclusion, to provide an accurate quantitative method that could assess the likelihood of 5‐year OS in BC, an effective predictive model was formulated by incorporating the four ARGs and two clinicopathological risk factors (age and TNM stage). Thus, this nomogram has the potential to identify the high‐risk patients and guide individualized efficient therapeutic strategies for BC patients.

## CONFLICT OF INTEREST

The authors declare that they have no competing interests.

## AUTHOR CONTRIBUTION

**Jianguo Lai:** Conceptualization (lead); Project administration (lead); Writing‐original draft (lead); Writing‐review & editing (lead). **Bo Chen:** Conceptualization (lead); Funding acquisition (lead); Project administration (lead); Writing‐original draft (lead); Writing‐review & editing (lead). **Hsiaopei Mok:** Data curation (lead); Resources (lead); Software (lead). **Guochun Zhang:** Formal analysis (lead); Investigation (lead); Supervision (lead). **Chongyang Ren:** Data curation (equal); Investigation (lead); Resources (lead); Software (equal); Visualization (lead). **Ning Liao:** Conceptualization (lead); Funding acquisition (lead); Project administration (lead); Writing‐original draft (lead); Writing‐review & editing (lead).

## Supporting information

Fig S1‐S4

## Data Availability

The data sets used and analysed during the current study are available from the corresponding author on reasonable request.
