# Supplementary material for: Comprehensive analysis of autophagy‐related prognostic genes in breast cancer
Source: J Cell Mol Med. 2020 Jul 2;24(16):9145–53. doi: 10.1111/jcmm.15551 (PMC7417718; doi:10.1111/jcmm.15551)

Supplementary Figure 1. The least absolute shrinkage and selection operator (LASSO) algorithms to identify the ARGs signature in the training cohort

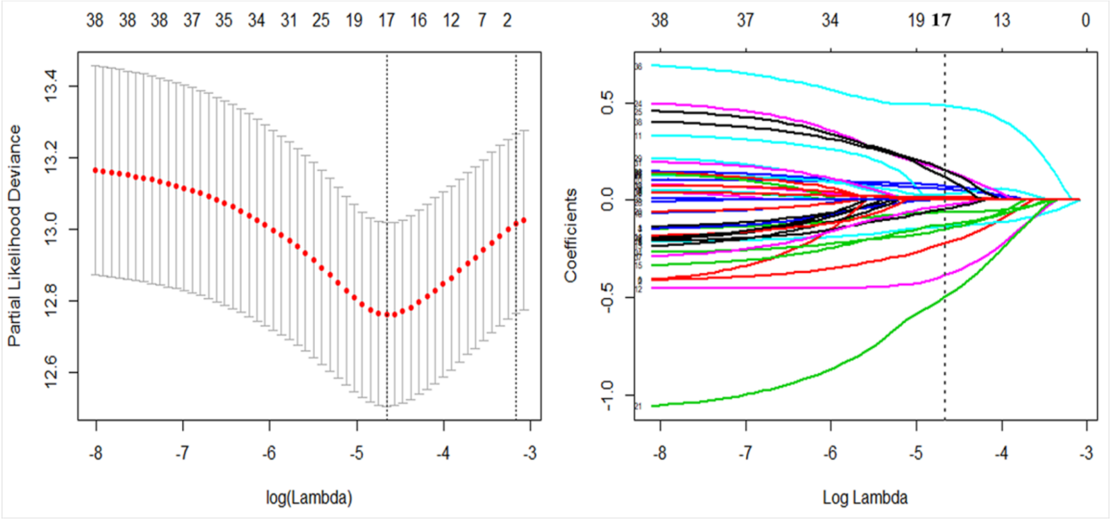

Supplementary Figure 2. Functional enrichment analysis of the 17 ARGs in the Metascape database **(A)** The 7 enrichment terms. **(B)** The network of the enrichment terms.

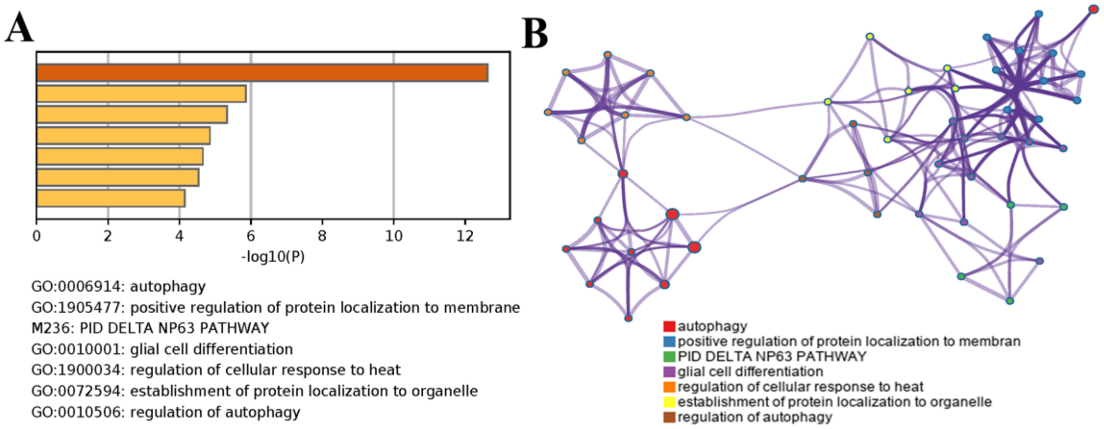

Supplementary Figure 3. Genetic alterations of the four ARGs in TCGA database.

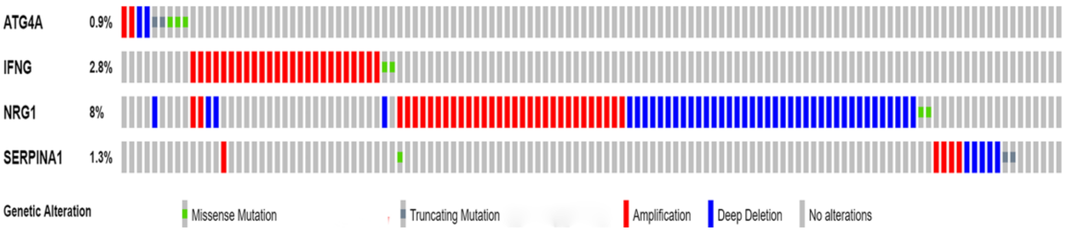

Supplementary Figure 4. Visualization of the relationship between the four ARGs and DNA methylation in TCGA database via the MEXPRESS tool

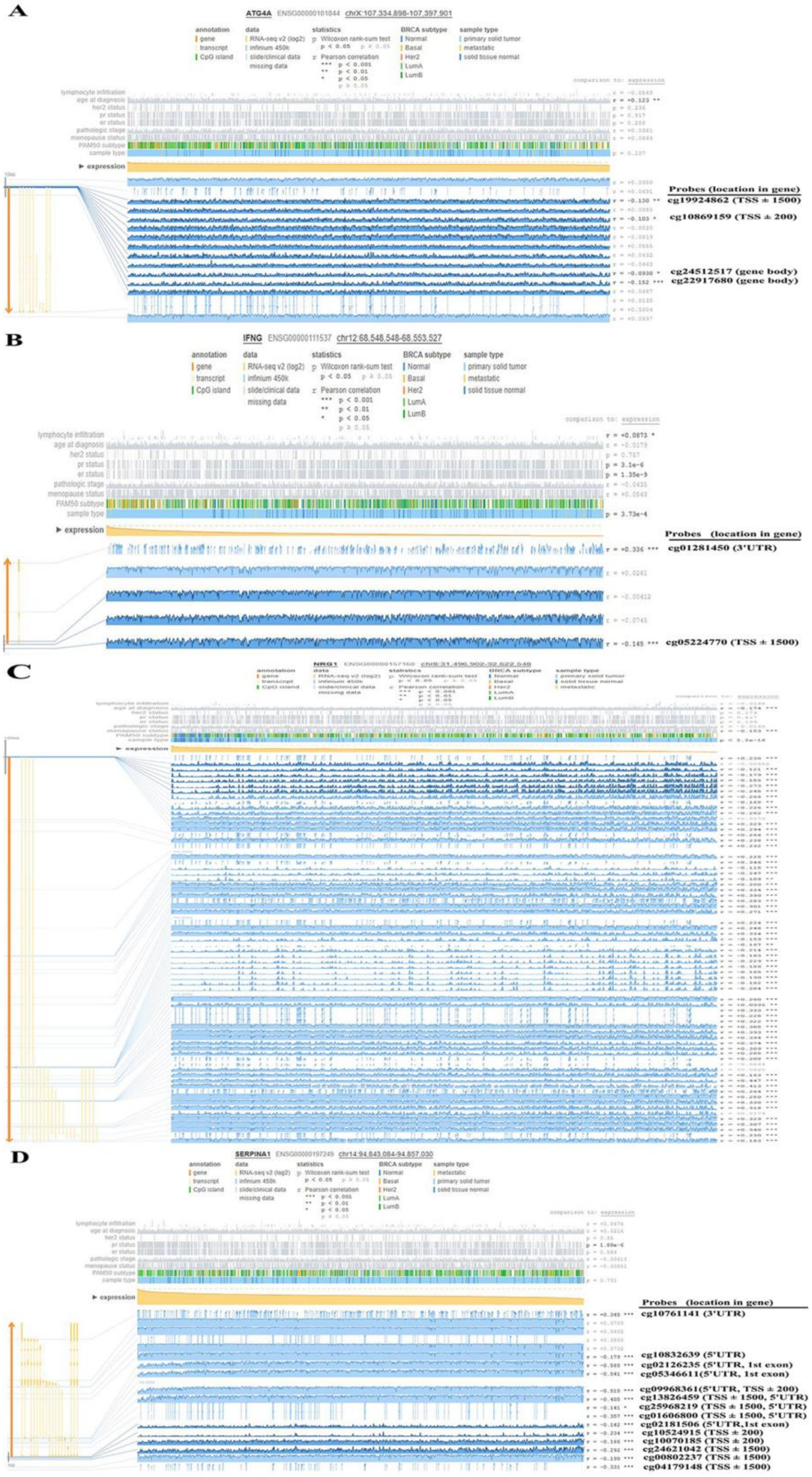

Supplement: Supplementary file 1 — Fig S1‐S4 [file JCMM-24-9145-s001.pdf]
